# Supplementary material for: Patterns of Intron Gain and Loss in Fungi
Source: PLoS Biol. 2004 Nov 30;2(12):e422. doi: 10.1371/journal.pbio.0020422 (PMC532390; doi:10.1371/journal.pbio.0020422)
Supplement: Table S1 — Also available at http://genes.mit.edu/NielsenEtAl/. (4.3 MB ZIP). [file pbio.0020422.st001.zip › NielsenEtAl/html/740.html]

AN9156.1.NCU09788.1.MG10669.1.FG09475.1


```
 CLUSTAL W (1.82) Multiple Sequence Alignments - Introns Inserted


Sequence 1: MG10669.1	618 aa
Sequence 2: FG09475.1	602 aa
Sequence 3: NCU09788.1	622 aa
Sequence 4: AN9156.1	600 aa
Alignment Length: 637 aa
Number Identitical Residues: 220 aa
Alignment Score (without introns) 12278


MG10669.1 	MLTNAPTAALVALVAGLVAPSL-AQSIAEINGNKFVSPLNDQDVKNVTGVVLAKGPNGIW
NCU09788.1	-----MKGVLCWAVAGLQPLLSAALTISEINGNRYISPYNGKTVSDVTGLVTAKSSAGIY
FG09475.1 	-------MKLQFLVSSLISPLVTALTIAEINGDRYLSPYQGKNVTAIEGLVTAVGSNGFY
AN9156.1  	-------MKIFATVLSLALPAA-AVTISEINGNAFLSPFNGESVSGVEGLVTAIGGEGFF
          	         :   * .*      * :*:****: ::** :.: *. : *:* * .  *::

MG10669.1 	LRSDKPDNDPLTSEAVYVYDSKVGANLAVGDLVALD-GRVLEYR~SDNKHLFLTEVTTTK
NCU09788.1	IRSTTPDDDEATSDSIYVFSSTIGRTVDVGDIINLGSAKVSEYR~SSNTYMYLTELTNPS
FG09475.1 	LRSTKPDRSAATSEGLYIFGKSAVGNVSVGDVVTLD-GFIEEYR2SNKDYIYLTELSSPK
AN9156.1  	LRSTNPDSDDATSESIYVYGNSSVSKVSVGDIITLS-GKVSEYR~SSDDYLYLTEITSPS
          	:** .** .  **:.:*::...   .: ***:: *. . : *** *.. :::***::...

MG10669.1 	NVRKLSSGNPAAPLVIGKDTSAPPTEQYTSLDDGDIFATPNAAARLTEVNPVLDPTKYGL
NCU09788.1	DITVVSSNNAVTPLVIGKDTLSPPTEQFTSLDEGDIYSVPNAQHNISAVNPVLKPAQYGL
FG09475.1 	NIVVKSSDNKFKPKVIGKDTGNPPGKEFSKLDDGNVFAVPNNKSLVSVANPKLQPNSYGL
AN9156.1  	SIVVKSSGNEVTPVVIGKDR-SPPTEEYSSLDTGDVLAVPNNVSQISVDNPVLQPDKYGM
          	.:   **.*   * *****   ** ::::.** *:: :.**    ::  ** *.* .**:

MG10669.1 	DFWESLNGELVTVRAPVALNRPNTYGDIWVAGDWPTTGRNARGGLTTLGVD~ANPEAILI
NCU09788.1	DFWESLTGELVTVKAPTALKIPSSYRDTWVIGDWKVTGKNDAGSLTMSAKD1SNPETILI
FG09475.1 	DFWESLSGELVTVSKAYALSRPNSFGDFWVRGNWKVSGLNKQGGLTMVGSD1ANPEAIVI
AN9156.1  	DFWESLSGELVSLTGVTLITKPNQYGDVFVRGDWAVTGLNGHGGLTQTEKD1SNPEAIKI
          	******.****::     :. *. : * :* *:* .:* *  *.**    * :***:* *

MG10669.1 	GTPLDGTKNPADTKLGDQMTEVTGVVTYAFGFYRILPLTAIRVATPIPFNPRNATLASTG
NCU09788.1	GAPLDSTKNPTTTKLGDALEDITGIVTNTYGFYTILPLTAIKIKTPSALTPPPTSLVSSG
FG09475.1 	GSPLDGTKNPDDTKLGDFVGEVTGVVTYAFGFYRILPLTAIKVSKRSNSEHPAVSFTSKG
AN9156.1  	GTPLDGTSNSDSSKVGDTVEDVTGVVQWNYGQYMVLPLTALKVTGSNDTTASPSALTGDG
          	*:***.*.*.  :*:** : ::**:*   :* * :*****:::           ::.. *

MG10669.1 	SCSGVTVGIFNVENLCPSSAHLPAIAAQLVDALKTPDLVFLQEVQDDSCATNDGVVDATN
NCU09788.1	SCNGLTVGTYNVENLAPTSAHMPKVASQIVKYLKSPDLLFIQEVQDNTGATDDGVVSANK
FG09475.1 	SCKGITVADYNTENLNPESAHLPLVVGQIVEKLRTPDLIFLQEVQDNSGAANDGVVSANK
AN9156.1  	TCEALAIGSYNVENLTPTSDNIEAIADHIANYLNGPAIMCLQEIQDNTGATDDGVVDANV
          	:*..:::. :*.*** * * ::  :. ::.. *. * :: :**:**:: *::****.*. 

MG10669.1 	TLEALRFAVSNLTDGKVDYSWVEVAPVDNQDGGQPGGNIRVAYLYKPSVLTLAEPTNSVG
NCU09788.1	TLATLISAIQSAGG--PTYDYVTIEPVNDEDGGQPGGNIRTAYLYNPSILSLKNPNP--G
FG09475.1 	TLAALADGIEAASG--VVYEWAEVVPDNNVDGGQPGGNIRQAYLYRPDVVQLVKPNE--G
AN9156.1  	TLSTLAELISAAGG--PDYDFTEIAPIDGEDGGEPGGNIRVAYLYDPTIVQLHNPNP--G
          	** :*   :.   .    *.:. : * :. ***:****** **** * :: * :*.   *

MG10669.1 	TGADANEVLPG-----PTLKFNPGRIDPANAAWAATRKPLVAAWKALNATKP-FFTVNVH
NCU09788.1	SSTDANEVIVDAETGQPSLKFNPGRVDPLNAAWRATRKPLVAEWIAKDGKKP-FFTVNVH
FG09475.1 	SASDVNAVLDG-----PTLKFNPGRIDPANSVWDNSRKPLAAEWKPVKGTKKSFFTVNVH
AN9156.1  	TSTDANEVQSG-----PELKYNPGLIDPTNEAWEASRKPLVAAWETVDGKNT-FYTINVH
          	:.:*.* *  .     * **:*** :** * .*  :****.* * . ...:  *:*:***

MG10669.1 	FSSKGGSSSLHGDLRTPDNSGVTKRTQQATVTG0EFIAKILAEDPEAQVIAAGDFNEFIP
NCU09788.1	MTSKGGGSSVHGDVRPPINGGVAKRTQQAEITA~KFISKILQADPAARVIAAGDFNEFAF
FG09475.1 	FGSKGGSSSIHGDARTPVNKGVEKRTKQSEVTA0DFVAQILKKDKKAHVIVAGDFNEFAD
AN9156.1  	FTSKGGGSYLQGDERPPVNGGVEQRTAQAEVVA0SFITSILEEDASAKILTTGDFNEFTF
          	: ****.* ::** *.* * ** :** *: :.. .*::.**  *  *:::.:******  

MG10669.1 	VSPLQTFMKVSGLLDLDEVVEMAPEERYSY~LFDMNTQQLDHTFVSPSLSVRTPCSRRSH
NCU09788.1	VKPLTTFAEVSGLKELDEALNTPVENRYTY2SYDMNAQALDHMYISPVLAS-----SKSK
FG09475.1 	VEPIDTFVKTSGLVDIDDAAKIPMTERYTY~LFDSNCQALDHMFISKELR------KGIK
AN9156.1  	AAPLKTFVSASGLQDLDEVVGVDPLERYTY~IYDSNHEQLDHMFVSEALA------EGAR
          	. *: ** ..*** ::*:.      :**:*  :* * : *** ::*  *          :

MG10669.1 	FQHLHVNTWSDTAGEVSDHDPSVGRFNVCA-----------
NCU09788.1	FQHIHVSSWVTDSAVVSDHDPSVALFDVCGGADSNKRVHKL
FG09475.1 	YEHMHINTWQDTAGEVSDHDPSVALFDLC------------
AN9156.1  	MEHVHVNTWVNYDDAPSDHDPSVAVLNVCE-----------
          	 :*:*:.:*       *******. :::*
```
